# Prescriber-level surveillance of outpatient antimicrobial consumption to enable targeted antimicrobial stewardship: a nationwide observational study, Switzerland, 2015 to 2022

**DOI:** 10.2807/1560-7917.ES.2024.29.37.2300734

**Published:** 2024-09-12

**Authors:** Sereina M Graber, Sabrina M Stollberg, Catherine Plüss-Suard, Carola A Huber, Andreas Kronenberg, Oliver Senn, Stefan Neuner-Jehle, Andreas Plate

**Affiliations:** 1 Department of Health Sciences, Helsana Group, Zurich, Switzerland; 2 Swiss Centre for Antibiotic Resistance (ANRESIS), Institute for Infectious Diseases, University of Bern, Bern, Switzerland; 3 Institute of Primary Care, University of Zurich and University Hospital Zurich, Zurich, Switzerland

**Keywords:** Antimicrobial consumption, outpatient setting, prescriber speciality, claims data, surveillance

## Abstract

**Background:**

In Europe and other high-income countries, antibiotics are mainly prescribed in the outpatient setting, which consists of primary, specialist and hospital-affiliated outpatient care. Established surveillance platforms report antimicrobial consumption (AMC) on aggregated levels and the contribution of the different prescriber groups is unknown.

**Aim:**

To determine the contribution of different prescribers to the overall outpatient AMC in Switzerland.

**Methods:**

We conducted a retrospective observational study using claims data from one large Swiss health insurance company, covering the period from 2015 to 2022. We analysed antibiotic prescriptions (ATC code J01) prescribed in the Swiss outpatient setting. Results were reported as defined daily doses per 1,000 inhabitants per day (DID) and weighted according to the total population of Switzerland based on census data.

**Results:**

We analysed 3,663,590 antibiotic prescriptions from 49 prescriber groups. Overall, AMC ranged from 9.12 DID (2015) to 7.99 DID (2022). General internal medicine (40.1% of all prescribed DID in 2022), hospital-affiliated outpatient care (20.6%), group practices (17.3%), paediatrics (5.4%) and gynaecology (3.7%) were the largest prescriber groups. Primary care accounted for two-thirds of the prescribed DID. Quantity and type of antibiotics prescribed varied between the prescriber groups. Broad-spectrum penicillins, tetracyclines and macrolides were the most prescribed antibiotic classes.

**Conclusion:**

Primary care contributed considerably less to AMC than anticipated, and hospital-affiliated outpatient care emerged as an important prescriber. Surveillance at the prescriber level enables the identification of prescribing patterns within all prescriber groups, offering unprecedented visibility and allowing a more targeted antibiotic stewardship according to prescriber groups.

**Key public health message**

**What did you want to address in this study and why?**

With increasing use, the effectiveness of antibiotics to tackle bacterial infections can be compromised as bacteria become drug resistant. Antimicrobial resistance contributes yearly to around 5 million deaths worldwide, and thus surveillance of antibiotic consumption, especially in the outpatient setting, is important. We wished to identify which group of physicians (primary care, specialists, hospital outpatient) prescribes the most antibiotics.

**What have we learnt from this study?**

Antibiotic prescriptions by 49 groups of physicians in the outpatient setting declined by 12.4% from 2015 to 2022 in Switzerland. With ca 66% in 2022, the largest prescriber group of primary care physicians (family medicine, general practice and internal medicine, gynaecology, paediatrics) contributed less than expected, whereas hospital-affiliated outpatient care was the second most important prescriber group with ca 20%.

**What are the implications of your findings for public health?**

The study shows that it is possible to analyse detailed antibiotic prescribing patterns on a prescriber level. This type of surveillance builds the basis for sharing information with specific groups of physicians, which can help to improve the responsible use of antibiotics and address the rise of resistance.

## Introduction

Rising rates of antimicrobial resistance (AMR) in bacteria pose an important public health threat [1,2] with antimicrobial consumption (AMC) being the main driver [3]. Knowledge of AMC is a requirement for antibiotic stewardship activities [2] and, like Switzerland, many countries have established respective surveillance platforms. The largest amount of antibiotics is prescribed in the outpatient setting [4-7], which includes primary care, specialist care and hospital-affiliated outpatient care [8]. For the Swiss outpatient setting, the national surveillance reported the consumption of 8.7 from a total of 10.1 (86%) defined daily doses (DDD) per 1,000 inhabitants per day (DID) in 2022 [9]. However, surveillance has thus far been carried out at an aggregate level, and neither Swiss national surveillance, the World Health Organization (WHO) nor the European Surveillance of Antimicrobial Consumption Network (ESAC-Net) report detailed antibiotic prescribing patterns on a prescriber level [4,5,10]. 

Available evidence suggests that the prescription of antibiotics in the outpatient setting varies across the different medical specialties [7,11,12]. Some reports on AMC originate from the United States (US) [6,11,13]. However, some important prescriber groups in the US, e.g. nurse prescribers, exist only in few European countries [14]. Consequently, findings from the US cannot directly be applied to the European context. In addition, there are varying measures for reporting AMC and the share of prescriptions does not automatically correspond to the share of consumption. The diversity of reporting measures limits comparison between countries or established surveillance platforms, which report AMC usually in DID [4,5].

To the best of our knowledge, a comprehensive overview on outpatient AMC that considers all prescriber groups has not been performed. Therefore, the primary objective of the study was to determine outpatient consumption of systemic antibiotics using established measures for AMC reporting and to provide details regarding the proportional contribution of the various prescriber groups to the overall AMC in the Swiss outpatient setting. Secondary objectives were to determine AMC patterns in the different prescriber groups and to determine characteristics of patients prescribed antibiotics.

## Methods

### Study design and setting

We conducted a retrospective observational study, using anonymised claims data from the Helsana Group, which is one of the largest health insurance companies in Switzerland, providing basic health insurance to ca 15% of the population.

The observation period was from 1 January 2015 to 31 December 2022. In Switzerland, a basic health insurance is mandatory for all residents. The choice of the provider, insurance model and deductible determine the premium. Independent of the provider, all basic health insurance plans cover the costs of all licensed and approved medications after the deductible and co-payment amounts have been paid. The outpatient setting in Switzerland does not distinguish between public and private healthcare sectors. 

For the analysis, we included all prescriptions from the outpatient setting. We excluded prescriptions from dentists, as dental procedures are usually not covered by basic health insurance and, as a result, prescriptions are either not submitted to health insurance at all or only very infrequently. We further excluded preparations marked as ‘investigational medicinal product’, sponges, rinse solutions, preparations for inhalations and for ocular use and prescriptions with incomplete dosing information. 

Antibiotic prescriptions were identified using WHO anatomical therapeutic chemical (ATC) code J01 [15]. Antibiotics in the WHO ATC groups P01AB (nitroimidazole derivatives) and A07AA (oral formulations of vancomycin and colistin) were reported separately. Prescriptions were grouped according to the 2023 WHO AWaRe classification [16], i.e. ‘Access’, ‘Watch’, ‘Reserve’. Patients of all ages were included. Patient’s chronic disease status was measured using pharmacy-based cost groups (PCG), which is a validated tool to map chronic conditions based on medication [17].

### Prescriber groups

Routine data from insurance claims comprise the information on medical groups, originally based on data by the imprest register [18]. In particular, each healthcare provider is required to report their respective specialisation or, in the case of multiple specialist titles, the medical field in which they primarily work. Data from different prescriber groups (n = 49) were analysed. Data on the following groups, which had the largest shares of prescriptions, were reported: (i) family medicine, general practice and internal medicine (referred hereafter as general internal medicine, GIM), (ii) hospital-affiliated outpatient care (HOS), (iii) otorhinolaryngology (ORL), (iv) urology (URO), (v) gynaecology (GYN), (vi) paediatrics (PED) and (vii) dermatology (DER). All other prescriber groups, including group practices as well as all other groups of specialists, were combined into one group (‘Others’). The GIM group comprises physicians who provide primary care services, excluding specialists in the different disciplines of internal medicine, e.g. cardiologists. These are categorised as separate prescriber groups. The HOS group encompasses all prescribers affiliated with hospitals, including outpatient clinics belonging to a hospital as well as discharge prescriptions for medication acquired in community pharmacies. The prescribers of the HOS group may be specialists from different fields, e.g. cardiologists, urologists. An overview of all analysed prescriber groups is given in Supplementary Table S1. 

We defined the prescriber groups GIM, PED and GYN as primary care [19]. ‘Group practices’ (classified as a prescriber within the ‘Others’ group) referred to practices with more than one prescriber group registered. For example, this prescriber group included large walk-in practices and the out-of-hours care services, with the majority of practices providing primary care services. Accordingly, we reported AMC for primary care with and without (conservative approach) group practices. Those prescribers who did not belong to primary care, HOS or group practices were referred to as specialists.

### Measurements of antimicrobial consumption

In general, by ‘consumption’, we refer to the antibiotics prescribed by individual healthcare provider groups. To measure AMC, we determined the number of individual antibiotic prescriptions and calculated the consumed DID. The DID were calculated by summing all prescribed DDD per ATC per year for the entire study population and extrapolating down to a daily dose of a population of 1,000 inhabitants. The prescribed number of DDD per prescription is calculated by dividing the total prescribed quantity of the substance (ATC) in grams by the corresponding DDD (version 2023) [15]. We reported antibiotic groups based on the classification used by the Swiss Centre for Antibiotic Resistance (ANRESIS) [4].

### Extrapolation

All numbers given represent weighted numbers to the total Swiss population (8.67 million, 2022 census). The weighting factors are given by the ratio between the Swiss and study population, stratified by year, region of residence (i.e. canton), sex and age as used in the risk equalisation statistics [17]. A detailed description of the method is found in Neuner-Jehle et al. [20]. This method reduces potential sociodemographic biases in the study population. Results of the main analysis based on raw data (not extrapolated) are provided in the Supplement. To estimate validity of our calculations, we compared AMC calculated in our study with AMC reported by ANRESIS (claims data vs sales-based data) [4].

### Statistics

We used descriptive statistics to report AMC, using the number of weighted prescriptions, weighted DID per year, ATC and prescriber group. Additionally, we calculated corresponding proportions, representing the share of each prescribers’ group in a given ATC group in a year. Patient characteristics represent extrapolated or weighted sociodemographic and morbidity characteristics of individuals who were prescribed at least one antibiotic per prescriber group per year. Either weighted means and interquartile ranges (IQR) or percentages are provided. To determine trends over time, we used the non-parametric Mann-Kendall Test. This test allows the assessment of whether a variable of interest is monotonically increasing or decreasing over time. Additionally, we calculated the Sen’s Slope – a non-parametric measure of the slope of a temporal trend – indicating the strength of a trend: a positive slope indicates an upward trend, and a negative slope indicates a downward trend.

## Results

We identified a total of 3,673,563 antibiotic prescriptions (non-extrapolated) during the whole observation period. After exclusion of 9,973 (0.0027%) prescriptions according to our exclusion criteria, we included 3,663,590 prescriptions in our final analyses.

### Overall antimicrobial consumption patterns

**Figure 1:**
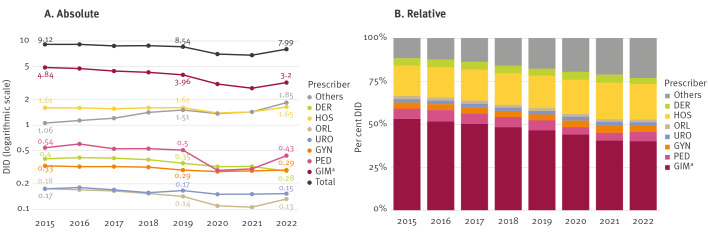
Absolute and relative contribution of the main prescriber groups to overall antimicrobial consumption in the outpatient setting per year, Switzerland, 2015–2022

DER: dermatology; DID: defined daily dose per 1,000 inhabitants per day; GIM: general internal medicine; GYN: gynaecology; HOS: hospital-affiliated outpatient clinics; ORL: otorhinolaryngology; PED: paediatrics; URO: urology.

^a^ GIM includes family medicine, general practice and internal medicine, excluding specialists in the different disciplines of internal medicine.

Overall outpatient AMC varied from 9.12 DID in 2015 to 7.99 DID in 2022 (relative decrease of 12.4%), with the lowest consumption rates occurring during 2020 and 2021, the years most affected by the COVID-19 pandemic, showing overall a significant decrease (p = 0.019; Sen’s Slope: −0.198). We have identified the largest prescriber groups as GIM with 3.2 DID (contribution of 40.1% to overall AMC in 2022), HOS with 1.65 DID (20.6%), and group practices (as a main part of the ‘Others’ group) with 1.38 DID (17.3%) (Figure 1, Table 1). Supplementary Table S1 provides the proportion of overall DID and the proportion of all antibiotic prescriptions for all prescriber groups for the years 2015 and 2022.

Numbers represent extrapolations for the entire Swiss outpatient setting.

**Table 1:** Overview of the 30 most important antibiotic prescriber groups to overall outpatient antimicrobial consumption, Switzerland, 2015 and 2022

Rank^a^	Prescriber group	2015	2022
1	General internal medicine	53.1%	40.1%
2	Hospital-affiliated outpatient clinics	17.6%	20.6%
3	Group practices^b^	4.7%	17.3%
4	Paediatrics	5.9%	5.4%
5	Gynaecology	3.6%	3.7%
6	Dermatology and venereology	4.4%	3.6%
7	Urology	1.9%	1.9%
8	Otorhinolaryngology	1.9%	1.7%
9	Pulmonology	1.0%	0.8%
10	Surgery	0.4%	0.5%
11	Gastroenterology	0.6%	0.4%
12	Rheumatology	0.8%	0.4%
13	Ophthalmology	0.4%	0.4%
14	Medical oncology	0.5%	0.3%
15	Cardiology	0.3%	0.3%
16	Orthopaedic surgery and traumatology of the musculoskeletal system	0.3%	0.3%
17	Plastic, reconstructive and aesthetic surgery	0.1%	0.2%
18	Maxillofacial surgery	0.2%	0.2%
19	Nephrology	0.2%	0.2%
20	Endocrinology and diabetes	0.4%	0.2%
21	Infectious diseases	0.1%	0.2%
22	Psychiatry and psychotherapy	0.3%	0.2%
23	Neurology	0.1%	0.2%
24	Allergology and clinical immunology	0.2%	0.2%
25	Physical medicine and rehabilitation	0.2%	0.2%
26	Haematology	0.2%	0.1%
27	Neurosurgery	< 0.1%	0.1%
28	Anaesthesiology	0.1%	0.1%
29	Child and adolescent psychiatry and psychotherapy	< 0.1%	< 0.1%
30	Angiology	0.1%	< 0.1%
Primary care setting^c^		67.3%	66.5%
Primary care setting (conservative approach)^c^		62.6%	49.2%

DID: defined daily doses per 1,000 inhabitants per day.

^a^ Ranked by contribution to overall antimicrobial consumption (overall DID) in 2022.

^b^ Practices with services of at least two different prescriber groups.

^c^ The primary care setting includes: general internal medicine (1), group practices (3), paediatrics (4) and gynaecology (5). In the conservative approach, prescriptions by group practices (3) were excluded.

Antimicrobial consumption is measured as per cent of total DID in a given year.

The relative contribution of the different prescriber groups to AMC changed during the observation period. We observed significant decreases in the relative contribution of the GIM (p < 0.001), and ORL (p = 0.009) groups, and a significant increase in the contribution of the HOS (p = 0.004) group, group practices and the prescribers summarised in the ‘Others’ group (each p < 0.001). The time trend analyses are provided in Supplementary Table S2. A total of 20% of all prescriptions in the HOS group were issued within a time frame of −2 days to +5 days following a hospital discharge. These prescriptions can be considered to be those issued following an inpatient treatment.

The number of prescriptions per 100 consultations decreased from 3.4 (2015) to 2.4 (2022). In 2022, urologists (9.1 prescriptions/100 consultations) and paediatrics (5.0/100) showed the highest prescription rates. The number of prescriptions per 100 insured persons ranged from 40.3 (2015) to 34.7 (2022). Supplementary Figure F1 shows the development of the number of prescriptions per 100 consultations and per 100 insured persons from 2015 to 2022. Within the 'Others' group, the predominant prescriber groups beside the group practices were pulmonology with 0.06 DID (0.8% of overall AMC in 2022), surgery with 0.04 DID (0.5%), and gastroenterology with 0.03 DID (0.4%); the data are provided in Supplementary Table S1.

Primary care contributed between 49.2% (conservative approach) to 66.5%, and specialist care to around 13%, to overall outpatient AMC in 2022. The relative contribution of primary care to overall outpatient AMC remained stable from 2015 to 2022 (67.3% vs 66.5%) and decreased AMC by GIM was equalised by increased AMC by group practices. The contribution in DID of the primary care sector from 2015 to 2022 can be found in Supplementary Table S3, the contribution of the specialist groups in Supplementary Table S1.

### Antimicrobial consumption of antibiotic substances

**Figure 2:**
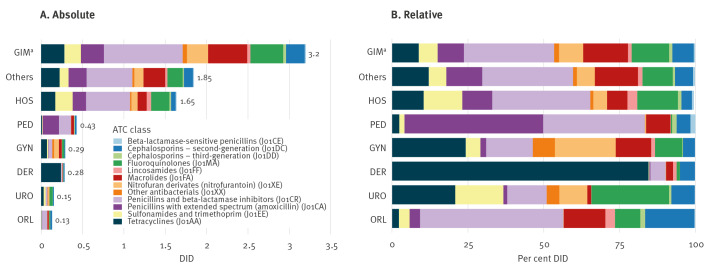
Absolute and relative antibiotic consumption by antibiotic class and prescriber group, Switzerland, 2022

ATC: anatomical therapeutic chemical; DER: dermatology; DID: defined daily dose per 1,000 inhabitants per day; GIM: general internal medicine; GYN: gynaecology; HOS: hospital-affiliated outpatient clinics; ORL: otorhinolaryngology; PED: paediatrics; URO: urology.

^a^ GIM includes family medicine, general practice and internal medicine, excluding specialists in the different disciplines of internal medicine.

Absolute and relative antibiotic consumption by prescriber group and antibiotic class in 2022 are presented in Figure 2. Supplementary Figure F2 provides the same information presented by antibiotic class and coloured by prescriber group. Penicillins in combination with a beta-lactamase inhibitor (J01CR, 29.1%, of which 99.96% were amoxicillin/clavulanic acid, J01CR02), tetracyclines (J01AA, 12.9%), and macrolides (J01FA, 11.8%) were the most prescribed antibiotic classes in 2022. Fluoroquinolones (J01MA, 11%) were ranked fourth. Relative contribution of antibiotic classes changed within the observation period. For example, we observed a stable consumption of amoxicillin/clavulanic acid (2.35 DID in 2015 vs 2.32 DID in 2022) but a small increase in the consumption of penicillins with extended spectrum (amoxicillin, J01CA: 0.69 DID in 2015 vs 0.87 DID in 2022). We found steady declines in the use of second-, and third-generation cephalosporin antibiotics and fluoroquinolone antibiotics (0.63, 0.16, 1.54 DID in 2015 vs 0.47, 0.08, 0.88 DID in 2022, respectively). Prescriptions of the different antibiotic classes changed over time and between the analysed prescriber groups. For example, we found that the amount of amoxicillin/clavulanic acid prescribed by paediatricians dropped by over 20% (0.19 DID in 2015 to 0.15 DID in 2022), whereas the overall consumption of that antibiotic class remained stable. In addition, we found the share of fluoroquinolones prescribed by the HOS group increased from 20.1% (2015) to 25% (2022), whereas in other groups such as GIM we observed a decrease of prescribing from 58.2% (2015) to 44.8% (2022). A full overview of AMC stratified by prescriber group and antibiotic class from 2015 to 2022 for all antibiotic classes with more than 0.01 DID is provided in Supplementary Table S4.

Numbers represent extrapolations for the whole of Switzerland.

Antimicrobial consumption of antibiotics in the P01AB and A07AA group were 0.14 DID and 0.12 DID in 2022. Most of the substances were prescribed by the GIM and HOS prescriber groups. The absolute numbers of DID by prescriber group over time and by antibiotic substance are provided in Supplementary Figure F3 and F4. The proportion of prescribed antibiotics in the ‘Access’ group increased from 53.9% in 2015 to 66.6% in 2022, whereas the proportion of the ‘Watch’ group declined from 45.7% (2015) to 33.2% (2022), and the ‘Reserve’ group stayed stable (2015: 0.06% to 2022: 0.08%). Supplementary Figure F5 provides the data according to the WHO AWaRe classification.

### Characteristics of antibiotic recipients

The mean age of a recipient of an antibiotic drug was 45.7 years (IQR: 40 years), 59.7% of patients were female, and 74.1% of antibiotic recipients were ensured in a managed care insurance model in 2022 (Table 2).

**Table 2:** Characteristics of patients prescribed a systemic antibiotic drug (J01) in the outpatient setting, Switzerland, 2022 (n = 1,674,493^a^)

Patient characteristics	Prescriber group								
	GIM	GYN	HOS	ORL	PED	URO	DER	Other	Total
Sociodemographic characteristics									
Mean age in years (IQR)	54 (35)	44.5 (26)	45.7 (41)	43.1 (33)	6.9 (7)	63.4 (22)	44.5 (36)	46.7 (36)	45.7 (40)
Male sex	37.8%	1.2%	45.2%	47.6%	50.6%	76.6%	40.5%	39.4%	40.3%
Female sex	62.2%	98.8%	54.8%	52.4%	49.4%	23.4%	59.5%	60.6%	59.7%
Ensured in a managed care insurance model	72.3%	78.6%	72.1%	71.5%	82.7%	66.6%	71.9%	73.3%	74.1%
Chronic diseases									
Cardiovascular	40.0%	24.8%	36.9%	30.0%	2.7%	55.0%	26.7%	33.4%	32.7%
Respiratory	20.5%	12.8%	17.2%	21.4%	19.2%	13.9%	12.5%	19.6%	18.1%
Diabetes	10.8%	6.4%	10.4%	6.6%	0.4%	14.8%	6.8%	9.4%	8.9%
Cancer	2.9%	2.4%	5.6%	2.3%	1.7%	5.2%	4.7%	3.3%	3.1%

DER: dermatology; GIM: general internal medicine; GYN: gynaecology; HOS: hospital-affiliated outpatient clinics; IQR: interquartile range; ORL: otorhinolaryngology; PED: paediatrics; URO: urology.

^a^ Data represent unique patients who received at least one J01 prescription in 2022.

The results show weighted (according to the whole Swiss population) sociodemographic and morbidity characteristics of individuals who were prescribed at least one antibiotic per prescriber group per year. Values are presented in percentage (%) if not stated otherwise. Patient characteristics for all years (2015–22) are provided in Supplementary Table S5.

Mean age (Sen’s slope: 0.3; p = 0.025), proportion of women (0.18; p = 0.029) and proportion of managed care insurance models (2.37; p < 0.001) increased significantly from 2015 to 2022. Prevalence of chronic diseases among antibiotic recipients varied between the different prescriber groups. In general, the prevalence of the considered chronic diseases was highest in the GIM, HOS and URO prescriber groups. During the years most affected by the COVID-19 pandemic (2020–21) we observed an increase in the mean age of antibiotic recipients. The proportion of patients with a respiratory comorbidity according to the evaluated PCG decreased during the pandemic years. In contrast, the proportion of patients with a cardiovascular disease, diabetes or cancer increased. The patient characteristics for all analysed years (2015–22) are provided in Supplementary Table S5.

### Explorative analysis

For most prescriber groups, the proportion of antibiotic prescriptions and the derived contribution to AMC (measured in DID) were very similar. However, some prescriber groups showed discrepancies comparing both measurements, e.g. paediatrics (contribution to overall prescribed DID vs contribution to all prescriptions: 5.4% vs 7.8%) or dermatology (3.6% vs 1.7%). Supplementary Table S1 shows the proportion of overall DID as well as the proportion of all prescriptions for all analysed prescriber groups. Comparing AMC calculations in this study to AMC reported by ANRESIS, we found slightly lower overall AMC in this study (range: overall AMC: −2.8% in 2017 to −8.1% in 2022; mean: −5.1%). Supplementary Table S6 shows the overall AMC of our study compared to overall outpatient AMC reported by the Swiss national surveillance from 2015 to 2022.

Comparing the prescriber groups GIM and group practices, we found that patients from the prescriber group of group practices were ca 10 years younger. In contrast, the distribution of female and male patients and the most common prescribed antibiotic substances were similar. Supplementary Table S7 shows the mean age, the proportion of female patients and number of prescriptions of antibiotics classes for the GIM and the ‘Others’ group.

Our analysis did not reveal any notable discrepancies in prescription patterns between the raw data and the extrapolated data (Supplementary Table S8 and S9, Supplementary Figure F6 and F7).

## Discussion

Our study determined the contribution of 49 different prescriber groups to total outpatient AMC. We found that GIM and HOS were the most important prescriber groups and primary care contributed only two-thirds to the overall outpatient AMC. We observed a decrease in total AMC from 2015 to 2022. Broad-spectrum penicillins were the most used antibiotics. The average recipient of an antibiotic prescription was a middle-aged female and up to one-third of patients had a chronic disease.

Primary care has always been considered the most important prescriber group in European countries and is usually attributed to up to 80% of all antibiotic prescriptions within the outpatient sector [7,12,21,22]. We found that primary care had contributed considerably less to Swiss outpatient AMC than anticipated. To date, most antibiotic stewardship interventions have primarily addressed the primary care setting. Our analysis revealed that other prescriber groups have also become important, and stakeholders need to recognise the relative importance of these prescriber groups in designing future antibiotic stewardship interventions.

Absolute AMC in the primary care sector has experienced a considerable decline. This decline is particularly notable when taking into account that the average number of patient consultations per Swiss primary care practice has increased by ca 15% from 2018 to 2022 but the number of primary care providers themselves remains largely unchanged [23]. However, within the primary care sector, there was a substantial rise in AMC issued by group practices. In contrast, relative AMC by the entire primary care sector remained unchanged. We explain this observation by the steady decrease in the number of individual practices and the steady increase in the number of group practices in Switzerland. Group practices largely comprise primary care providers, encompassing not only in-hours and continuity of care but also walk-in clinics offering out-of-hours care. Therefore, they may have a higher proportion of younger patients and possibly more consultations because of acute infections [24]. Accordingly, these prescribers and patients represent an important target group for future antibiotic stewardship interventions.

Hospital-affiliated outpatient clinics were the second largest prescriber group, accounting for ca 20% of all outpatient AMC. The importance of HOS in the Swiss outpatient setting was previously unrecognised. In comparison, an English surveillance programme reported that only ca 7% of AMC in the outpatient setting could be attributed to the hospital-affiliated outpatient clinics [7]. A recent publication reported inappropriate prescribing rates in ca 43% of hospital-affiliated outpatient clinics [25], which is comparable to the rates of up to 50% reported in primary care [26]. Given that the main focus of hospital-based antibiotic stewardship initiatives to date has been on inpatient use of antibiotics [27], future antibiotic stewardship efforts will require stakeholders to consider the specifics of hospital-based outpatient care.

Although our data showed a reduction in overall outpatient AMC, divergent patterns emerged among prescriber groups and antibiotic classes. Surveillance at the prescriber level also allows for the detection of anomalous patterns. For example, we found that the decrease in fluoroquinolone use in the HOS group was less than in other groups. Whether this reflects the required level of appropriate prescriptions in this clinical setting, or represents ongoing inappropriate use remains unclear and should be evaluated in further studies.

Knowledge about basic characteristics of antibiotic recipients is important to improve antibiotic consumption. Characteristics of antibiotic recipients in this study were similar to characteristics reported in other studies analysing AMC in the Swiss primary care setting [28]. The present study provides evidence of the extent to which the presence of comorbidities was unequally distributed between the prescribers. This link is important since the presence of comorbidities is a known risk factor for antibiotic use [29]. Our data confirmed sex differences [29,30], with women accounting for ca 60% of all prescriptions. 

To the best of our knowledge, AMC surveillance at the prescriber level has not yet been established in any European country. In Switzerland, information on prescribing patterns beyond the aggregated data was not yet accessible and prescriber groups were unaware of their prescribing patterns. Our approach allows for detailed analysis of all single prescriber groups. Our analysis may encourage stakeholders in countries where routine data, such as insurer data, can identify individual prescribers and thus affiliation to a particular prescriber group to expand their own surveillance to the prescriber level. In the best-case scenario, monitoring AMC at prescriber level can become part of national surveillance systems.

In addition to overall AMC trends, positive and negative trends at the substance class level among individual prescriber groups, which were previously concealed in the overall aggregated reporting, are now visible. This information can be crucial to a more comprehensive allocation of antibiotic stewardship campaigns’ efforts, both for primary care and medical specialists. While the specialties' contribution to overall AMC is limited, optimising antibiotic use at all levels is necessary. One initial antibiotic stewardship intervention may be providing feedback to the various medical societies and specialist groups, with the aim of increasing awareness of the prescribing behaviour of its own group (aggregated and anonymised).

To the best of our knowledge, our study provides the first comprehensive overview of the relative contribution of all prescriber groups to an overall AMC in an outpatient setting. Our calculations are, in terms of overall consumption across all prescriber groups, similar to the aggregated data published by the national surveillance, highlighting the accuracy of our findings. The slightly lower estimates of ca 5% can be attributed to the different data source (claims vs sales data). Using insurer data has the advantage of providing information on the quantity of prescriptions issued by specific prescribers and the corresponding amount of antibiotics issued. In contrast to sales data analysis, which may have included antibiotics that are stored in warehouses and are still to be dispensed to patients, we could analyse prescriptions that were actually handed out to the patients. We could show that for some prescriber groups, relevant differences in the relative contribution to all antibiotic prescriptions and to the overall antibiotic consumption emerged. For example, in dermatology or gastroenterology, we found that the proportion of overall prescribed DID was considerably greater than the proportion of the number of antibiotic prescriptions. This might be explained by the prolonged therapies in these medical specialities. In the context of AMC surveillance, one needs to be aware of these differences for certain prescriber groups.

Our study has limitations. Firstly, we could only include antibiotic prescriptions that were reported. Insurance companies are not aware of prescriptions obtained out-of-pocket for antibiotics. Although unknown, we consider this share to be minimal because the compulsory health insurance typically covers antibiotics. Secondly, unlike in sales data analysis, we could not evaluate prescriptions made by dentists. Dentists represent a significant prescriber group [31,32]. Dentists in Switzerland regularly prescribe antibiotics and a representative national survey revealed that 4% of the population had used antibiotics for dental infections within the previous 12 months [33,34]. Their absolute contribution to outpatient AMC remains unclear. Prescription data from dentists are included in the national surveillance data. Consequently, the observed difference between our calculations and the national surveillance can provide an indication of the extent of prescriptions by dentists. Thirdly, the calculations in this study are likely to slightly overestimate the true AMC because it is not possible to distinguish between antibiotics obtained by patients and those actually taken. Finally, Switzerland is a country with one of the lowest AMC compared with other European nations [4]. Prescribing behaviour and the structure of the outpatient setting itself may differ from other countries. Therefore, the findings of this study may not be applicable to every European country.

## Conclusion

Our findings highlight the advantages of comprehensive knowledge of antibiotic prescribing patterns at the prescriber level. Primary care in Switzerland contributed considerably less to the overall AMC than initially anticipated, whereas outpatient care affiliated to hospital visits emerged as an important prescriber group. Surveillance of AMC at the prescriber level enables the identification of prescribing patterns within all prescriber groups, offering unprecedented visibility. These monitoring results can be shared on an aggregated and anonymised level in a targeted manner according to prescriber groups to help guide specific antibiotic stewardship interventions.
